# Identification of a Novel Linear B-Cell Epitope of HbpA Protein from *Glaesserella parasuis* Using Monoclonal Antibody

**DOI:** 10.3390/ijms24108638

**Published:** 2023-05-09

**Authors:** Geyan Liu, Kang Wang, Zhen Yang, Xiaoyu Tang, Yung-Fu Chang, Ke Dai, Xinwei Tang, Bangdi Hu, Yiwen Zhang, Sanjie Cao, Xiaobo Huang, Qigui Yan, Rui Wu, Qin Zhao, Senyan Du, Xintian Wen, Yiping Wen

**Affiliations:** 1 Research Center of Swine Disease, College of Veterinary Medicine, Sichuan Agricultural University, Chengdu 611130, China; 2019303062@stu.sicau.edu.cn (G.L.); wangkkang156@163.com (K.W.); yzhen@stu.sicau.edu.cn (Z.Y.); xiaoyutang1@outlook.com (X.T.); daike1990@stu.sicau.edu.cn (K.D.); xinweitang1998@163.com (X.T.); hbd18982172170@outlook.com (B.H.); zhangyiwen@stu.sicau.edu.cn (Y.Z.); csanjie@sicau.edu.cn (S.C.); huangxiaobo@sicau.edu.cn (X.H.); yqg@sicau.edu.cn (Q.Y.); wurui1977@sicau.edu.cn (R.W.); zhao.qin@sicau.edu.cn (Q.Z.); senyandu@163.com (S.D.); xintian@sicau.edu.cn (X.W.); 2 Department of Population Medicine and Diagnostic Sciences, College of Veterinary Medicine, Cornell University, New York, NY 14850, USA; yc42@cornell.edu

**Keywords:** *Glaesserella parasuis*, HbpA protein, monoclonal antibody, antigen epitopes

## Abstract

*Glaesserella parasuis* (*G. parasuis.*) is the etiological pathogen of Glässer’s disease, which causes high economic losses to the pig industry. The heme-binding protein A precursor (HbpA) was a putative virulence-associated factor proposed to be potential subunit vaccine candidate in *G. parasuis.* In this study, three monoclonal antibodies (mAb) 5D11, 2H81, and 4F2 against *recombinant HbpA (*rHbpA*)* of *G. parasuis* SH0165 (serotype 5) were generated by fusing SP2/0-Ag14 murine myeloma cells and spleen cells from BALB/c mice immunized with the rHbpA. Indirect enzyme-linked immunosorbent assay (ELISA) and indirect immunofluorescence assay (IFA) demonstrated that the antibody designated 5D11 showed a strong binding affinity with the HbpA protein and was chosen for subsequent experiments. The subtypes of the 5D11 were IgG1/κ chains. Western blot analysis showed that mAb 5D11 could react with all 15 serotype reference strains of *G. parasuis*. None of the other bacteria tested reacted with 5D11. In addition, a linear B-cell epitope recognized by 5D11 was identified by serial truncations of HbpA protein and then a series of truncated peptides were synthesized to define the minimal region that was required for mAb 5D11 binding. The 5D11 epitope was located on amino acids 324-LPQYEFNLEKAKALLA-339 by testing the 5D11 monoclonal for reactivity with 14 truncations. The minimal epitope 325-PQYEFNLEKAKALLA-339 (designated EP-5D11) was pinpointed by testing the mAb 5D11 for reactivity with a series of synthetic peptides of this region. The epitope was highly conserved among *G. parasuis* strains, confirmed by alignment analysis. These results indicated that mAb 5D11 and EP-5D11 might potentially be used to develop serological diagnostic tools for *G. parasuis*. Three-dimensional structural analysis revealed that amino acids of EP-5D11 were in close proximity and may be exposed on the surface of the HbpA protein.

## 1. Introduction

*Glaesserella parasuis* (*G. parasuis*) is a non-motile, pleomorphic, nicotinamide adenine dinucleotide (NAD)-dependent gram-negative bacterium of the *Pasteurellaceae* family [1], which is a commonly found opportunistic parasitic pathogen in the upper respiratory tract of swine [2]. The original name of the bacterium was *Haemophilus Parasuis* (*H. parasuis*), but in 2020, Dickerman et al. analyzed the phenotypic characteristics and whole genome sequence of the bacterium and confirmed that *H. parasuis* was distinct from other genera in the family *Pasteurellaceae* and proposed to have it renamed as *Glaesserella parasuis* [3]. At present, at least 15 serotypes of *G. parasuis* have been identified, plus several non-typeable (NT) isolates, while the pathogenicity level of different serotypes is different [4]. Furthermore, the dominant serotypes are different in different regions [5]. Under some specific circumstances, including poor feeding and management practices, stress, and hypo-immunity, virulent strains breach the mucosal barrier and enter the bloodstream, causing a serious tissue and organ disease, called Glässer’s disease, which induces a strong inflammatory response characterized by meningitis, pneumonia, polyarthritis, and fibrinous polyserositis, leading to high morbidity and mortality in piglets, resulting in major economic losses in the global pig industry [4,6].

Meanwhile, Glässer’s disease tends to occur following infection by viral or other bacterial pathogens [7]. In such cases, it makes diagnosis and treatment of the pathogens a challenge, highlighting the need for discriminating diagnostic methods. Monoclonal antibodies with a high affinity and specificity for bacterial protein could be used to detect *G. parasuis* and precisely diagnose Glässer’s disease by immunological methods [8]. Screening of immunodominant antigens and epitope mapping is considered a crucial element in designing a targeted immune response, screening promising subunit vaccine candidates, and developing diagnostic methods [9]. The monoclonal antibody of *G. parasuis* Fe(3+) ABC transporter substrate-binding protein has also been demonstrated for the prevention and control of *G. parasuis* infection, including eliminating the bacteria in the blood and provided protection against *G. parasuis* by passive immunization protection experiments [10]. Mapping antigenic determinants are essential for the development of epitope-based serological diagnostic tools to assess antigen–antibody interactions for various diseases. The monoclonal antibody of *G. parasuis* OppA protein has also been proven to be helpful in dot blot and Western blot for reactivity with 1–15 serotype reference strains and could be used to develop serological diagnostic tools. Furthermore, a highly conserved linear B-cell epitope (aa 469-KTPAEAR-475) recognized by the mAb was identified by a phage-displayed 12-mer random peptide library and alignment analysis, which was highly specific among different bacterial strains [8]. Precise analysis of the epitope will provide the essential information for the development of diagnostic tools for *G. parasuis*.

The *hbpA* gene of *G. parasuis* encodes a peptide ABC transporter substrate-binding protein, which is also heme-binding protein A precursor (HbpA), and has high homology with *Haemophilus influenza* (*H. influenzae*) *hbpA* gene (also known as *gbpA*) [11]. *H. influenzae* HbpA has turned out to be a conserved and heme-dependent protein among *Haemophilus* species [12], implicated in the acquisition and utilization of heme into an organism as an iron source, and is considered to be a crucial element for the survival of the bacteria in the host because of the absolute growth requirement [13]. However, Vergauwen et al. reported that the dominant function of HbpA is mediating glutathione transport rather than heme import [11], which reflects the multiple functions of the substrate binding protein. The *hbpA* was identified as a virulence determinant in this model of *H. influenzae* invasive disease [14]. In addition, the recombinant protein HbpA could induce high titers of antibodies and mediate opsonophagocytosis, but vaccination with HbpA protein individually elicited low protective immunity against *Actinobacillus pleuropneumoniae* [15]. HbpA protein has also been identified in *G. parasuis* serotype 5 strain SH0165 [16], and is considered to be a putative virulence-related factor by analyzing the expression differences of membrane proteomes between two different virulence strains [17]. At the same time, mice immunized with this protein could produce high titers of antibodies, which could provide beneficial immune protection against 5 × LD_50_ (6 × 10^9^ CFU) *G. parasuis* M-3 strain, with 70% protection rate in mice [18]. Another report showed that this protein had a 40% protection rate against the *G. parasuis* high virulence SH0165 strain with LD_100_ (2.0 × 10^9^ CFU) *G. parasuis* lethal challenge in mice [19]. Therefore, the dominant epitope region of the protein can be screened to enrich the *G. parasuis* epitope map and develop diagnostic methods based on the epitope or a peptide vaccine against *G. parasuis* infection.

In our study, we described the generation and epitope mapping of *G. parasuis* mAb 5D11 and investigated the conserved epitopes among *G. parasuis* without reactivity with other bacteria. Three mAbs against purified recombinant *G. parasuis* HbpA protein were prepared, and the mAb 5D11 was found to exhibit the highest reactivity with HbpA protein by ELISA and IFA and was selected for further study of the antigenic epitope identification. In addition, mAb 5D11 could recognize *G. parasuis* in Western blot. Given that there may be additional applications of mAb 5D11, this study aimed to characterize the epitope that mAb 5D11 binds. An integrated approach was employed, including truncated protein expression and peptide synthesis. The epitope (EP-5D11/325-PQYEFNLEKAKALLA-339) was highly conserved among *G. parasuis*, which was confirmed by alignment analysis. Furthermore, 3D structure analysis showed that amino acids of EP-5D11 were in close proximity and may be exposed on the surface of the HbpA protein. Detailed information related to the EP-5D11 will assist us by providing us with a better ability to combat this disease and facilitate the development of diagnostic methods in the future.

## 2. Results

### 2.1. Expression, Purification, and Characterization of Full-Length G. parasuis Recombinant HbpA Protein (rHbpA)

**Figure 1:**
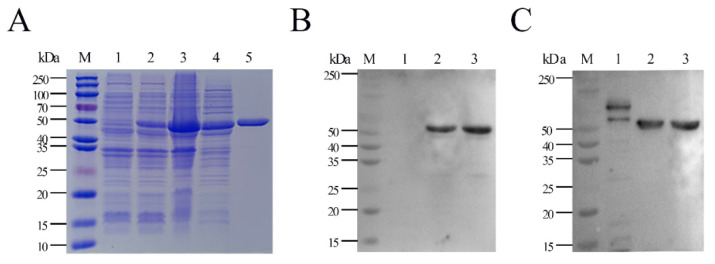
Expression and purification of His-tagged *G. parasuis* rHbpA protein. (**A**) SDS-PAGE of pET28a-HbpA transfected *E. coli* BL21(DE3). M: protein molecular weight marker; lane 1: IPTG-induced *E. coli* BL21(DE3) with empty vector pET-28a (+); lane 2: IPTG-induced *E. coli* BL21(DE3) with pET-28a-HbpA; lane 3: precipitates of pET28a-HbpA *E. coli* BL21(DE3) post sonication; lane 4: supernatant of pET28a-HbpA *E. coli* BL21(DE3) post sonication; lane 5: purified rHbpA protein (dialyzed, ultra-filtrated). (**B**) Western blot of *E. coli* BL21(DE3) with empty vector pET-28a (lane 1) and expressed vector pET28a-HbpA (lane 2,3) probed with mouse anti-His mAb (1: 5000). (**C**) Western blot of *E. coli* BL21(DE3) with empty vector pET-28a (lane 1) and expressed vector pET28a-HbpA (lane 2, 3) probed with mouse anti-GPS hyperimmune serum (1:200).

rHbpA protein was expressed as a fusion protein with its C-terminal and N-terminal 6×His tags, facilitating the purification process using Ni affinity chromatography in vitro. The products were dialyzed, ultra-filtrated, and analyzed by utilizing 12.5% sodium dodecyl sulfate polyacrylamide gel electrophoresis (SDS-PAGE, Figure 1A), while the *E. coli* BL21 strain with empty vector pET-28a(+) served as a negative control (lane 1). The expressed GPS-rHbpA was soluble in the supernatant post sonication (lane 4), and lanes 5 show the Ni-NTA purified rHbpA at the expected size, 55 kDa, indicating that the rHbpA protein was successfully expressed in BL21(DE3) under the induction by 1 mM of IPTG for 16–18 h at 26 °C. The Western blot (Figure 1B,C) demonstrated that the rHbpA protein was recognized explicitly by the mouse anti-His mAb at 1:5000 dilutions and the mouse anti-GPS hyperimmune serum at 1:200 dilutions.

### 2.2. Production and Screening of G. parasuis rHbpA mAbs

**Figure 2:**
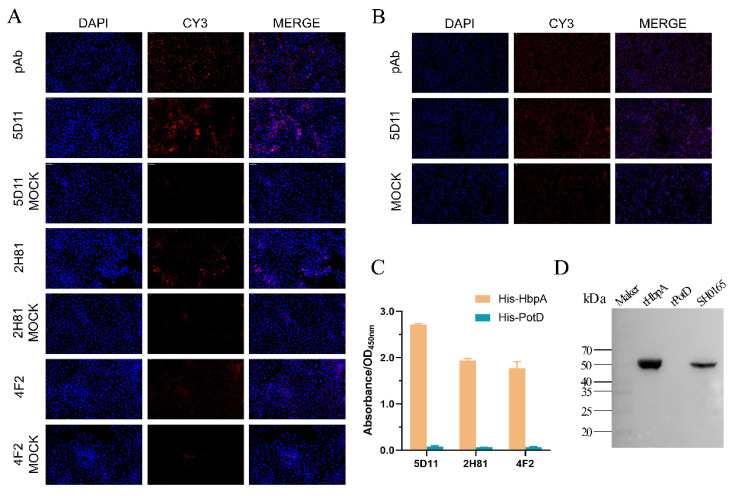
The activity of anti-GPS rHbpA mAbs. (**A**) Immunofluorescence assay (IFA) of the three mAbs reactivity with the *G. parasuis* in infected PK-15 cells. Binding was visualized with CY3 labeled goat anti-mouse antibody, while DAPI was used to visualize the cell nuclei. The mAb used for the assay is indicated on the left. Magnification = 200×. (**B**) Immunofluorescence assay (IFA) of mAb 5D11 reactivity with the *G. parasuis* in infected 3D4/21 cells. Magnification = 100×. (**C**) Activity of the antibodies with His-HbpA and His-PotD by ELISA. (**D**) Reaction of mAb 5D11 with rHbpA, rPotD, and *G. parasuis* SH0165.

Purified *G. parasuis* rHbpA was used to immunize BALB/c mice; a week after the booster immunization, the mouse with the highest antibody titer was sacrificed for hybridoma production. Hybridomas were screened four times by indirect ELISA, and ultimately, three hybridomas (named 5D11, 2H81, and 4F2, respectively) were chosen for further testing. The hybridomas were identified as IgG1 k light chain isotype using a mouse monoclonal antibody isotyping ELISA kit (Proteintech, Chicago, IL, USA). As can be seen from the representative indirect immunofluorescence assay (IFA) images (Figure 2A), the monoclonal 5D11 showed the most significant reactivity with the *G. parasuis* in infected PK-15 cells. Meanwhile, in infected 3D4/21 cells (Figure 2B), mAb 5D11 performed better in *G. parasuis* recognition than PK-15 cells. ELISA results (Figure 2C) also demonstrated that mAb 5D11 had greater reactivity with the rHbpA than the other mAbs. As seen in the Western blot results (Figure 2D), mAb 5D11 could recognize both the natural HbpA protein in *G. parasuis* and the rHbpA protein expressed in *E. coli* BL21(DE3) system, while rPotD protein expressed in the same vector, pET-28a(+), was served as a negative control.

### 2.3. Epitope Mapping of G. parasuis rHbpA

**Figure 3:**
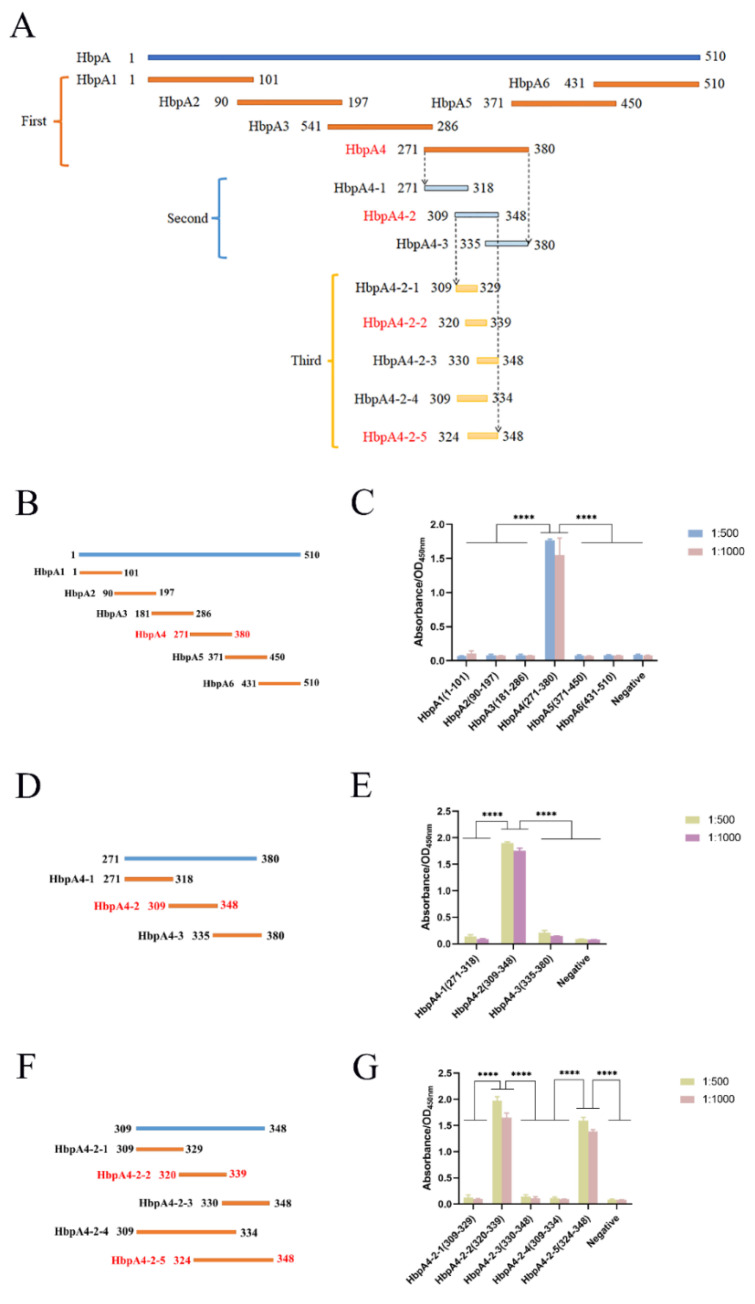
*G. parasuis* HbpA protein epitope mapping. The segments recognized by mAb 5D11 are marked in red and those unrecognized by 5D11 are marked in black. (**A**) Schematic of *G. parasuis* HbpA protein epitope mapping. The truncated fragments in the first round are represented by orange lines, the second round by blue lines, and the third round by yellow lines. (**B**,**C**) HbpA protein epitope mapping and ELISA for the reactivity of mAb 5D11with truncated segments (the first round). (**D**,**E**) HbpA4 epitope mapping and ELISA for the reactivity of mAb 5D11with truncated segments (the second round). (**F**,**G**) HbpA4-2 epitope mapping and ELISA for the reactivity of mAb 5D11with truncated segments (the third round). All ELISA experiments were performed in triplicate, and dilution ratios of monoclonal 5D11 are shown in the upper right corner. **** *p* value < 0.0001.

**Figure 4:**
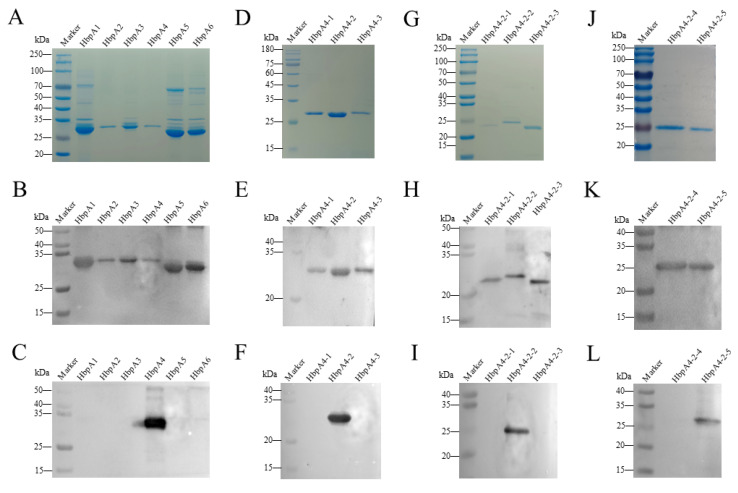
*G. parasuis* HbpA truncated expression and epitope mapping. (**A**,**D**,**G**,**J**) SDS-PAGE of the HbpA protein truncations expressed in *E. coli* BL21(DE3). (**B**,**E**,**H**,**K**) Western blot of truncated fragments probed with mouse anti-His mAb (1:5000). (**C**,**F**,**I**,**L**) Western blot of truncated fragments probed with mAb 5D11 (1:500).

Figure 3A shows the schematic diagram of protein segmentation in three rounds. To locate the epitope on HbpA recognized by mAb 5D11, IEDB Analysis Resource online analysis software (http://tools.immuneepitope.org/main/) was used to predict. Based on the B-cell epitope analysis results, we divided the HbpA protein into six parts ((Figure 3B): HbpA1 (1-101 aa), HbpA2 (90-197 aa), HbpA3 (181-286 aa), HbpA4 (271-380 aa), HbpA5 (371-450 aa), and HbpA6 (431-510 aa)). ELISA (Figure 3C) and Western blot (Figure 4C) results showed that mAb 5D11 reacted only with the HbpA4 fragment. From here, three truncations of the HbpA4 fragment were constructed ((Figure 3D): HbpA4-1 (271-218 aa), HbpA4-2 (309-348 aa), and HbpA4-3 (335-380 aa)). ELISA (Figure 3E) and Western blot (Figure 4F) showed that mAb 5D11 reacted only with the HbpA4-2 fragment. Then, we divided the HbpA4-2 fragment into five parts ((Figure 3F): HbpA4-2-1 (309-329 aa), HbpA4-2-2 (320-339 aa), HbpA4-2-3 (330-348 aa), HbpA4-2-4 (309-334 aa), and HbpA4-2-5 (324-348 aa)). ELISA (Figure 3G) and Western blot (Figure 4I) showed that mAb 5D11 reacted with the HbpA4-2-2 and HbpA4-2-5 fragments. These results demonstrated that the amino acids 324–339 were, at a minimum, necessary for the 5D11 interaction. Except for HbpA1, HbpA3, HbpA5, and HbpA6, all truncated proteins were soluble (Figure 4A,D,G,J). All proteins were expressed with His-tag, and Western blot results demonstrated that all truncated ones were specifically recognized by His-tag mAb (Figure 4B,E,H,K).

### 2.4. Identification of the Minimal Epitope

**Figure 5:**
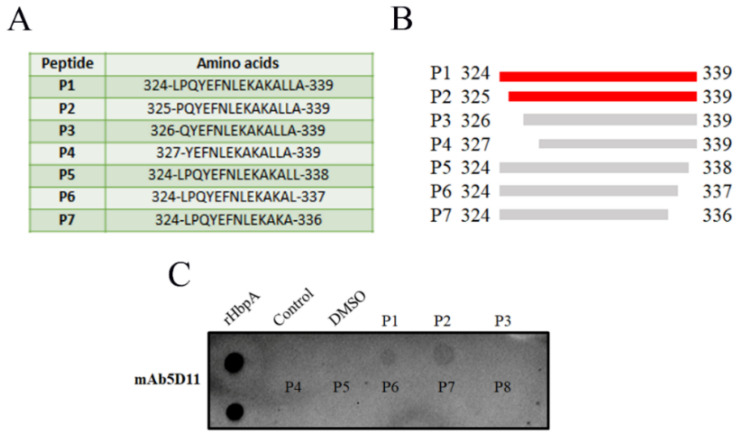
Identification of the minimal 5D11 epitope. (**A**) Seven peptides synthesis (P1, P2, P3, P4, P5, P6, P7). (**B**) Seven peptides reacted with mAb 5D11 to detect the minimum epitope. The peptides recognized by mAb 5D11 are represented by red lines, while those unrecognized by monoclonal 5D11 are represented by gray lines. (**C**) Seven peptides were tested for reactivity with mAb 5D11 by dot blot assays. The experiments were repeated three times.

The synthesized peptides (Figure 5A,B) were used as antigens in dot blot assays; the result showed that mAb 5D11 reacted most strongly with P1 and P2 and did not react with other peptides (Figure 5C), demonstrating that the minimal epitope recognized by mAb 5D11 was amino acids 325–339.

### 2.5. Cross-Reactivity Analysis

**Figure 6:**
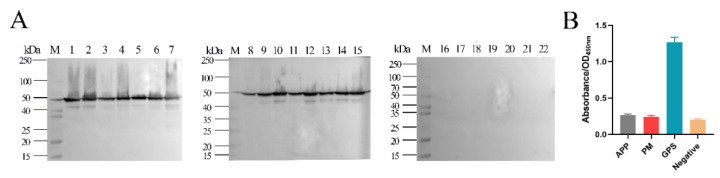
Identification specificity of mAb 5D11 by Western blot and indirect ELISA. (**A**) Reactivity of mAb 5D11 with the lysates of 15 serotype reference strains of GPS (lane 1-15), APP (lane 16), PM (lane 17), SS (lane 18), SC (lane 19), SA (lane 20), ETEC (lane 21), and ER (lane 20). The mAb 5D11 was used as the primary antibody. (**B**) Reactivity of EP-5D11 with mouse anti-APP, anti-PM, and anti-GPS hyperimmune. The negative control was nonimmunized mice serum.

Western blot was performed to investigate whether GPS (*G. parasuis*) cross-reacted with APP, PM, SS, SC, SA, ETEC, and ER on the epitope of EP-5D11 (aa 325-PQYEFNLEKAKALLA-339). mAb 5D11 was used as the primary antibody. As shown in Figure 6A, mAb 5D11 reacted with all 15 serotype reference strains of *GPS* but failed to react with others. A broad band with an approximate molecular mass of 50 kDa was also observed. This result showed that *GPS* EP-5D11 had no cross-reaction with APP, PM, SS, SC, SA, ETEC, and ER. 

### 2.6. Alignment Analysis

**Figure 7:**
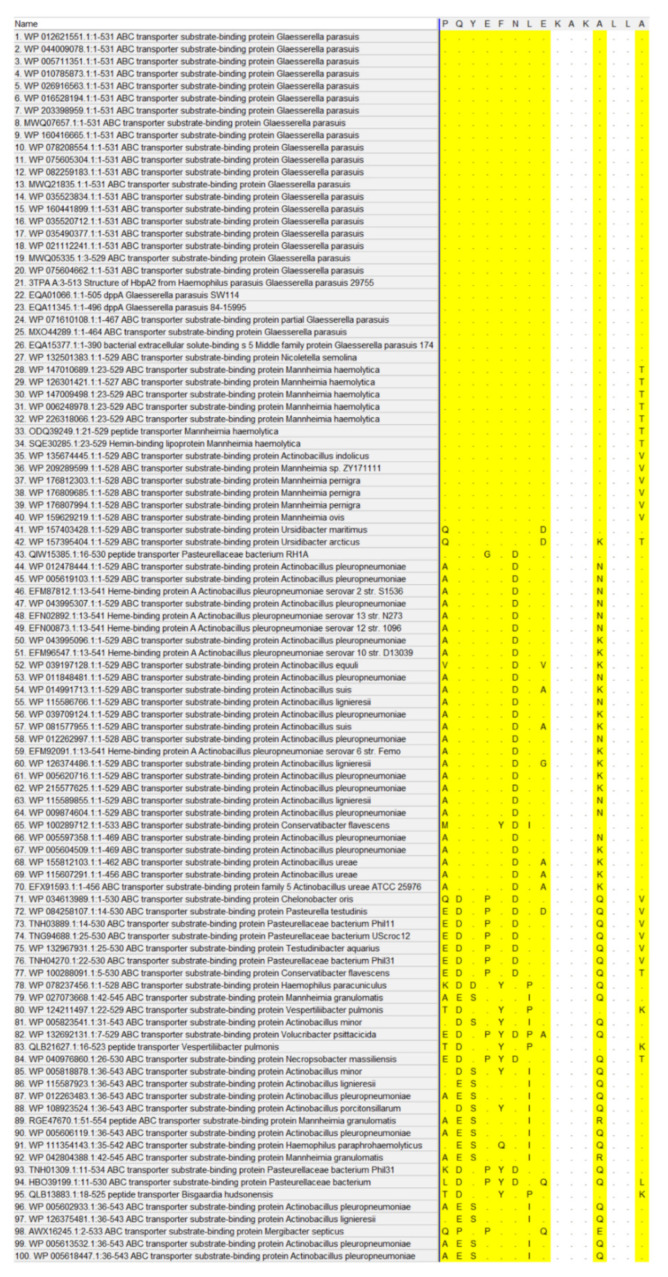
Comparison of the EP-5D11 amino acid sequence among different strains. The sequence corresponding to the region encompassing *G. parasuis* minimal linear epitope was aligned. The homologous sequences of different strains corresponding to the identified epitope are highlighted, where different amino acids are shown, and the same are replaced by dots. The first 26 were sequence alignments of *G. parasuis* from GenBank. The rest were sequence alignments of the epitope region from other animal bacteria. The GenBank accession numbers of strains were shown in front of the strains’ names. The results were analyzed by MEGA.

To explore the conservation of the EP-5D11 (epitope 5D11), we aligned the identified epitope with proteins available in GenBank. EP-5D11 was compared in NCBI BLAST and the sequence alignment results were displayed by MEGA (Figure 7). The alignment result showed that the EP-5D11 (aa325-PQYEFNLEKAKALLA-339) was highly conserved among the *G. parasuis* strains analyzed, with a shared sequence similarity of 100% (the first 26 in Figure 7). Meanwhile, the results revealed that *Nicoletella semolina*, *Mannheimia haemolytica*, *Mannheimia pernigra*, *Ursidibacter maritimus*, *Ursidibacter arcticus*, and *Actinobacillus indolicus,* share high sequence similarity in the position of epitope 5D11. Among them, *Nicoletella* and *Mannheimia* mainly infected horses [20], cattle [21] and sheep [22], and *Ursidibacter* from the bear mouth [23]. The APP shares 80% sequence similarity with *G. parasuis* in epitope 5D11, having three different amino acids at P^325^, N^330^, and A^336^. The PM share 60% sequence similarity with *G. parasuis* in epitope 5D11, having six different amino acids at P^325^, Q^326^, E^328^, N^330^, L^331^, and A^336^. EP-5D11 conjugated KLH as an antigen to detect the reaction with mouse anti-APP, anti-PM, and anti-GPS hyperimmune serum by indirect ELISA, as shown in Figure 6B; the positive mouse serum of APP and PM showed no cross-reaction with EP-5D11.

### 2.7. Three-Dimensional Structure Analysis of EP-5D11

**Figure 8:**
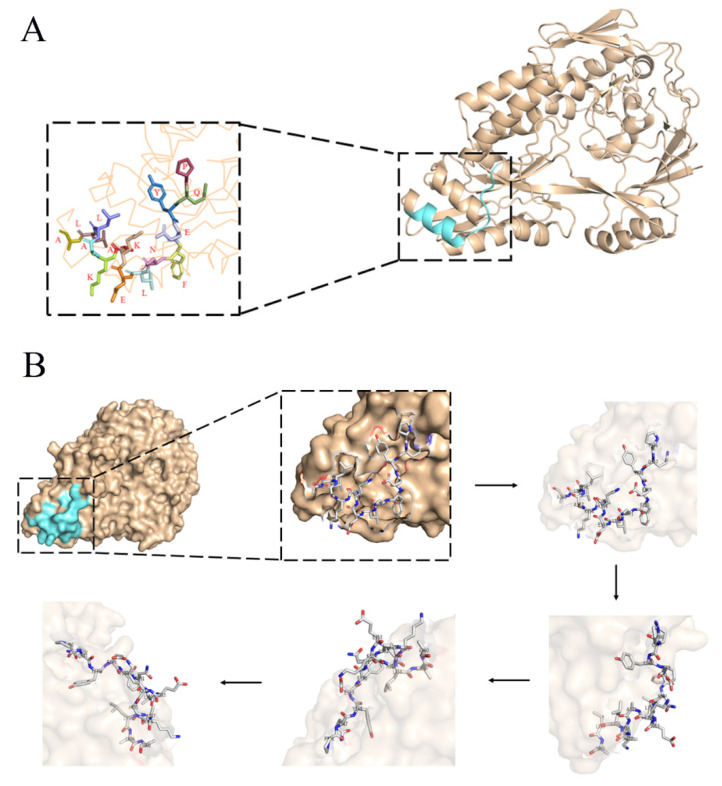
The 3D structure of the HbpA protein was visualized using the PyMOL molecular graphics and modeling system. (**A**) The overall structure of the HbpA protein is shown on the right, where the blue areas represent EP-5D11 residues (aa 325-PQYEFNLEKAKALLA-339), magnified on the left. (**B**) The EP-5D11 residues are shown as a stick figure and are displayed at different angles of rotation.

PyMoL molecular visualization system was used to analyze EP-5D11 from the 3TPA.1 (*G. parasuis* 29755 strain) template of the protein database (PDB). The amino acids of EP-5D11 were located close to one and were predicted to be exposed on the surface of the HbpA protein (Figure 8), suggesting that EP-5D11 was highly likely to be a linear epitope.

## 3. Discussion

*G. parasuis* is a widespread symbiosis in the benign porcine upper respiratory tract which may cause severe vascular lesions and multiple organ dysfunction, resulting in serious economic losses [6]. Monoclonal antibodies are widely used in the detection of microorganisms, such as viruses [24], bacteria [25], and other pathogens [26]. Since the critical factor in preparing monoclonal antibodies is to obtain a high purity immune antigen, the preparation method of bacterial-related monoclonal antibodies generally starts from the preparation of antigen protein [10]. In this study, soluble *G. parasuis* rHbpA protein was successfully expressed and used as an immunogen to immunize BALB/C mice. Monoclonal antibodies against *G. parasuis* rHbpA protein were generated by fusing spleen cells from BALB/c mice with SP2/0 murine myeloma cells. Three hybridoma cells (named 5D11, 2H81, and 4F2, respectively) were screened and preliminarily identified by indirect ELISA and IFA. The results demonstrated that hybridoma cell 5D11 had greater reactivity with the rHbpA than the other mAbs, and then it was selected to analyze the conserved and novel immune epitope of *G. parasuis*.

Identification of B-cell mapping epitopes with recognition function region by monoclonal antibodies is the basis for the development of epitope-based diagnostic tools, epitope vaccines, and therapeutic antibodies [10]. Several monoclonal antibodies against *G. parasuis* proteins have been reported, such as outer membrane protein A (OmpA) [27], Oligopeptide permease A (OppA) [8], and Fe(3+) ABC transporter substrate-binding protein [10], which were generated by fusing spleen cells from BALB/c mice immunized with the whole bacterial cells with SP2/0 murine myeloma cells. However, based on our knowledge, only OppA was identified in the relative region and mapped the precise location recognized by mAb 1B3, which was a highly conserved linear B-cell epitope matched ^469^KTPAEAR^475^ contributing to the cross-reaction between *G. parasuis* and other animal bacteria. There are many methods for epitope identification, among which functional methods to detect the binding activity of antigen fragments or synthetic peptide with antibody are easy to operate and widely utilized [28]. After three rounds of protein expression, fourteen truncations of HbpA were constructed: HbpA1 (1-101 aa), HbpA2 (90-197 aa), HbpA3 (181-286 aa), HbpA4 (271-380 aa), HbpA5 (371-450 aa), HbpA6 (431-510 aa), HbpA4-1 (271-218 aa), HbpA4-2 (309-348 aa), HbpA4-3 (335-380 aa), HbpA4-2-1 (309-329 aa), HbpA4-2-2 (320-339 aa), HbpA4-2-3 (330-348 aa), HbpA4-2-4 (309-334 aa), and HbpA4-2-5 (324-348 aa). Here, the epitope region recognized by mAb 5D11 was determined by serially truncating the HbpA protein (aa324-LPQYEFNLEKAKALLA-339). From there, for further precise mapping, a series of progressively truncated peptides were synthesized and served as antigens in dot blot assays, thus determining the minimal determinant of the mAb 5D11-binding site as aa325-PQYEFNLEKAKALLA-339 (named EP-5D11). Western blot analysis indicated that mAb 5D11 reacted with all 15 serotype reference strains of *G. parasuis* but not with other non-*G. parasuis* bacteria, such as APP, PM, SS, SC, SA, ETEC, and ER (Figure 5A). These results suggested that the epitope recognized by mAb 5D11 might be a species-specific epitope, which could be used to distinguish *G. parasuis* from other bacteria. In addition, sequence alignments of EP-5D11 demonstrated that the motif was highly conserved among *G. parasuis* strains. Furthermore, we found that the EP-5D11 sequence similarity was also extremely high with *Nicoletella semolina*, *Mannheimia haemolytica*, *Mannheimia pernigra*, *Ursidibacter maritimus*, *Ursidibacter arcticus*, *Actinobacillus indolicus.* The APP shares 80% sequence similarity with *G. parasuis* in epitope 5D11, having three different amino acids at P^325^, N^330^, and A^336^. The PM shared 60% sequence similarity with *G. parasuis* in epitope 5D11, having six different amino acids at P^325^, Q^326^, E^328^, N^330^, L^331^, and A^336^. Among them, *Nicoletella* and *Mannheimia* mainly infected horses [20], cattle [21] and sheep [22], and *Ursidibacter* from the bear mouth [23]. However, *Actinobacillus indolicus* [29], APP [30], and PM [31] can infect pigs. The mouse anti-APP and anti-PM hyperimmune serum were verified with no cross-reaction with EP-5D11. However, it remains to be further studied whether EP-5D11 can cross-react with *Actinobacillus indolicus*, due to a lack of positive serum. EP-5D11 was highly conserved among *G. parasuis* strains and was specific among different bacterial strains, suggesting that it could be developed as a potential diagnostic antigen for *G. parasuis* and other animal bacteria.

To our knowledge, this is the first report to identify the *G. parasuis* HbpA protein antigenic epitope EP-5D11 (aa 325-PQYEFNLEKAKALLA-339). It is a linear B-cell epitope and highly conserved among *G. parasuis* strains. In summary, we developed a mAb 5D11 and defined the highly conserved linear B-cell epitope within HbpA protein. The *G. parasuis* specific-mAb 5D11 and its defined linear epitope EP-5D11 could be used to develop *G. parasuis* epitope-associated diagnostics and vaccine design.

## 4. Materials and Methods

### 4.1. Ethics Statement

The handling of animals and all procedures were conducted in strict accordance with a protocol approved by the Institutional Animal Care and Use Committee of Sichuan Agricultural University, Sichuan, China (protocol code SYXK2019-187) and complied with the Guide for the Care and Use of Laboratory Animals of the Ministry of Science and Technology of the People’s Republic of China. Adult female-specific pathogen-free (SPF) BALB/c mice weighing 20–25 g (6–8 weeks old) were obtained from Dossy Experimental Animal Co., Ltd., Chengdu, China. Our experimental protocols were designed to provide comfort and minimal stress for our experimental mice.

### 4.2. Strains, Bacterial Growth Conditions, Plasmids, Cells, and Primers

*G. parasuis* SH0165 (GPS) and *Pasteurella multocida* HN-06 (PM) were kindly supplied by Xuwang Cai and Bin Wu from Huazhong Agricultural University, respectively, China. *Salmonella choleraesuis* (SC) was kindly supplied by Xinxin Zhao from Sichuan Agricultural University, China. The reference strains of *G. parasuis* (strains 1 to 15), *Actinobacillus pleuropneumoniae* (APP), *Enterotoxigenic Escherichia coli* (ETEC), *Streptococcus suis* (SS), *Staphylococcus aureus* (SA), and *Erysipelothrix rhusiopathiae* (ER) were preserved by the Laboratory of Research Center of Swine Disease in Sichuan Agricultural University. *Escherichia coli* DH5α (Biomed, Beijing, China) and BL21(DE3) (Biomed, Beijing, China) were cultured in liquid Luria-Bertani (LB, BD-Difco, NJ, USA) medium or on LB agar (LA, BD-Difco, NJ, USA) plates. *G. parasuis* was grown in Tryptic Soy Broth (TSB, BD-Difco, NJ, USA) or on a Tryptic Soy agar (TSA, BD-Difco, NJ, USA) plate, supplemented with 5% inactivated bovine serum (Solarbio, Beijing, China) and 0.1% (*w*/*v*) nicotinamide adenine dinucleotide (NAD, Sigma-Aldrich, Maryland, USA) (TSB++ and TSA++). When necessary, the media was supplemented with 50 µL kanamycin (Kan, 100 mg/mL) or 100 µL ampicillin (Amp, 100 mg/mL). All strains were shaken at 220 r/min (1 r = 2πrad) at 37 °C. The standard *E. coli* expression vectors, pET-28a(+) and pET-32a(+), were preserved by the Laboratory of Research Center of Swine Disease in Sichuan Agricultural University. Sp2/0-Ag14, PK-15, and 3D4/21 cells preserved by the Laboratory of Research Center of Swine Disease in Sichuan Agricultural University were maintained at 37 °C in a humidified 5% CO_2_ atmosphere in Dulbecco’s modified Eagle medium (DMEM; Gibco, Carlsbad, CA), supplemented with 10% heat-inactivated fetal bovine serum (PAN-Biotech, Aidenbach, Germany).

### 4.3. Expression/Purification of Full-Length and Truncated Recombinant HbpA Protein

Expression of recombinant protein of HbpA (rHbpA) was performed using an *E. coli* expression system. Briefly, PCR fragments containing *G. parasuis hbpA* gene minus a 21 amino acid signal peptide sequence were amplified from genomic DNA of *G. parasuis* SH0165 using primers HbpA-F and HbpA-R (Table 1) designed with Primer 5.0. The resulting 1575 bp PCR products were cloned into the restriction enzyme sites *Bam*HI and *Hin*dIII of linearized pET-28a (+) using ClonExpress II One Step Cloning Kit (Vazyme, China), giving rise to pET-*hbpA*. The integrity of the resulting construct (pET-*hbpA*) was verified by restriction enzyme digestion and DNA sequencing. When reaching an optical density at 600 nm (OD_600nm_) of 0.5 to 0.6, *E. coli* BL21(DE3) bearing pET-*hbpA* was induced to express by the addition of 1 mM isopropyl-β-D-thiogalactopyranoside (IPTG) for 16–18 h at 26 °C. The culture was collected by centrifugation at 5000× *g* for 10 min, resuspended in 50 mM Tris-HCl, lysed by ultrasonication, and then centrifuged again at 12,000× *g* for 10 min to pellet cell debris; the His-tag fusion recombinant HbpA protein was purified from the supernatant post sonication by Ni-NTA His-Bind Resin (5 mL prepacked column, Bio-Rad, Boulder, CO, USA), according to the instructions. Purified rHbpA was dialyzed with 2 L of PBS for 2 days and then analyzed by SDS-PAGE electrophoresis. Using the same methodology, a series of truncated HbpA proteins were produced, including HbpA1, HbpA2, HbpA3, HbpA4, HbpA5, HbpA6, HbpA4-1, HbpA4-2, HbpA4-3, HbpA4-2-1, HbpA4-2-2, HbpA4-2-3, HbpA4-2-4, and HbpA4-2-5, among which HbpA1, HbpA2, HbpA3, HbpA4, HbpA5, and HbpA6 were induced to express by the addition of IPTG for 16–18 h at 26 °C, while others were induced at 18 °C.

### 4.4. Western Blot

Western blot was used to test the reactivity of mouse anti-His mAb and mouse anti-GPS hyperimmune serum against the rHbpA protein. Purified rHbpA and *E. coli* BL21(DE3) with empty vector pET-28a(+) were subjected to 12.5% SDS-PAGE and then electrotransferred onto a 0.22 μm PVDF membrane. The membrane was blocked with 5% skim milk in TBST at room temperature (RT) for 2h and then incubated with mouse anti-His mAb (1: 5000) or mouse anti-GPS hyperimmune serum (1:200) overnight at 4 °C. The membrane was washed three times with TBST and then incubated with HRP-conjugated goat anti-mouse IgG (1:5000) at RT for 1 h. Finally, the membrane was washed four times with TBST, and the reaction results were visualized using enhanced chemiluminescence reagents (ECL; Bio-Rad, Boulder, CO, USA).

Western blot was also carried out to detect the specificity of mAb 5D11. The lysates of all 15 serotype reference strains of GPS and other seven species (APP, PM, SS, SC, SA, ETEC, and ER) were subjected to SDS-PAGE. The mAb 5D11 (1:500) was used as the primary antibody.

Western blot was also used to detect the reactivity of mAb 5D11 with truncations of the HbpA protein. To identify whether the epitope recognized by mAb 5D11 was linear, the truncated proteins were denatured, as previously described [28]. Briefly, the truncated proteins were mixed with 6× protein-loading buffer with DTT (TransGen Biotech, Beijing, China) and then heated for 8 min at 95 °C; this procedure fully denatured the secondary structure. Then, the truncations of the HbpA protein were subjected to SDS-PAGE and were tested with the mAb 5D11 (1:500) or mouse anti-His mAb (1:5000) using Western blot.

### 4.5. Production and Subtype Identification of Anti-rHbpA Protein mAbs

Monoclonal antibodies were prepared for epitope mapping. Anti-rHbpA protein mAbs were produced as follows: Six-week-old female BALB/c mice purchased from Chengdu Dossy Experimental Animal Co, Ltd., Chengdu China, were inoculated via subcutaneous injection with purified rHbpA protein (100 µg/mouse) mixed with an equal volume of Montanide Gel 01 PR adjuvant (Montanide, SEPPIC, Puteaux, France). Mice were boosted twice, at 2-week intervals, with the same immunogen and adjuvant. Pre-immune serum samples were taken from all mice and tested for reactivity against rHbpA by indirect ELISA. Three days before cell fusion, mice with the highest antibody titer were inoculated via subcutaneous injection with only purified rHbpA protein (100 µg/mouse). Three days after the final boost, mice were sacrificed, and spleens removed. Next, splenocytes were fused with Sp2/0 Ag14 cells using polyethylene glycol (PEG 1500; Sigma, Ronkonkoma, NY, USA). Following fusion, hybridomas were diluted into 96-well plates and cultured in hypoxanthine-aminopterin-thymidine (HAT)-DMEM selection medium. After 7 days, half of the medium was removed and replaced with fresh HAT-DMEM medium. After 13 days, the medium was removed and replaced with a hypoxanthine-thymidine (HT)-DMEM medium. Reactive hybridomas were subcloned four times by limiting dilution, and culture supernatants from individual hybridoma clones were screened for reactivity and specificity with rHbpA by indirect ELISA.

Eight-week-old BALB/c mice were injected intraperitoneally with hybridoma 5D11, and ascitic fluid was collected and purified using the octylic acid ammonium sulfate method (CA-AS). Subtype classes of mAbs were identified using a Mouse Monoclonal Antibody Isotype ELISA Kit (Proteintech, Chicago, IL, USA), according to the manufacturer’s protocol. This approach produced one mAb against rHbpA protein, named mAb 5D11.

### 4.6. Indirect ELISA

Indirect ELISA was performed to test the reactivity of culture supernatants from individual hybridoma clones against the rHbpA protein. Briefly, the wells of a 96-well ELISA plate were coated with 1 µg/well of rHbpA protein overnight at 4 °C. The next day, the wells were rinsed three times with PBST (1× PBS with 0.05% Tween 20) and then blocked with 5% (*w*/*v*) skim milk (PBST as diluent) for 2 h at 37 °C (200 μL/well). Next, the wells were rinsed three times with PBST and then incubated with the supernatant of hybridoma culture for 1 h at 37 °C (100 μL/well). At the same time, the serum from immunized and nonimmunized mice served as either positive or negative controls. Then, wells were rinsed again and incubated with HRP-conjugated goat anti-mouse IgG (1:5000, Sigma, USA) for 30 min at 37 °C (100 μL/well). After the final rinsing, wells were incubated with 100 μL tetramethylbenzidine (TMB, Tian Gen, Tianjin, China) for 15 min, and color development was stopped with 50 μL 2M H_2_SO_4_. Finally, the OD_450_ was read by an ELISA plate reader (Bio-Rad, Boulder, CO, USA). When the ratio of the positive value (P) to the negative value (N) was greater than 2.1 (P/N > 2.1), the result was judged positive.

Indirect ELISA was also carried out to detect the reactivity of mAb 5D11 with truncations of the HbpA protein. The wells of a 96-well ELISA plate were coated with 1 µg/well of truncated proteins as antigens. The mAb 5D11 (1:500) and negative controls were used as primary antibodies. Other operations were as described above.

Keyhole limpet hemocyanin (KLH) was selected as the carrier protein. The identified EP-5D11 conjugated KLH was synthesized (Sangon Biotech, Shanghai, China) at 1 mg/tube. The EP-5D11 conjugated KLH was coated with 2 µg/well. The mouse anti-APP, anti-PM, and anti-GPS hyperimmune serum were used as primary antibodies to detect the reactivity with EP-5D11.

### 4.7. IFA

In order to verify whether mAb 5D11 could recognize the *G. parasuis* in infected cells, IFA was used. Sterile coverslips were placed into the bottom of 12-well plates and then inoculated with PK-15 cells and 3D4/21 cells (80% well confluency). Half the wells were inoculated with *G. parasuis* SH0165 at a dose of 10 MOI (1 × 10^6^ cells vs. 1 × 10^7^ bacteria), and half were mock inoculated and then the cell plate was incubated at 37 °C for 2 h. After post-infection, cells were washed three times with PBS and fixed with 4% formaldehyde for 30 min. The supernatant of hybridoma culture (1:25) or mouse anti-GPS hyperimmune serum (1:100) were used as the primary antibodies, and goat anti-mouse IgG/CY3 antibody (1:500 in PBST, Sigma, USA) was used as the secondary antibody. Nuclei were stained with DAPI (Solarbio, Beijing, China).

### 4.8. Identification of the Minimal 5D11 Epitope

To determine the minimal B-cell epitope recognized by mAb 5D11, seven peptides spanning various lengths of aa 324–339 were commercially synthesized (GenScript, Nanjing, China). The peptides were synthesized with standard F-moc-solid phase peptide synthesis (GenScript, Nanjing, China). According to an assessment with high-performance liquid chromatography (HPLC), the purity of the peptides was >95%. The amino acid sequence of these peptides (P1–P7) is shown in Figure 4A,B. As previously described, the reactivity of mAb 5D11 with the peptides was tested by dot-blotting. Specifically, peptides were dissolved in DMSO (Solarbio, Beijing, China) to a concentration of 10 mg/mL for dot-blotting, and 1 µL (10 µg) was spotted on the PVDF membrane.

### 4.9. Dot Blot Analysis

Dot-blot hybridization for the identification of linear epitopes was based on the method described by Chen et al. 2017 [32]. Briefly, PVDF membranes were soaked in dimethyl sulfoxide and then in methanol. A total of 1 µL (0.5 µg) of proteins and 1 µL (10 µg) of peptides were spotted onto the activated membrane and then air-dried for 10 min at room temperature. Protein denaturation reagents were omitted during sample preparation to preserve the native conformation in the dot blot. After blocking with 2% BSA in TBST for 30 min at room temperature, mAb 5D11 was added in 2% BSA/TBST and incubated at 37 °C for 1 h. Next, the membrane was rinsed and then incubated with HRP-conjugated goat anti-mouse IgG (1:5000) for 1 h at 37 °C. Finally, the membrane was washed four times with TBST, and the reaction results were visualized using ECL.

### 4.10. Alignment Analysis

In order to determine the specificity and conservation of the identified epitope by mAb 5D11 among *G. parasuis* strains, we aligned the identified epitope with proteins available in GenBank and analyzed it by MEGA.

### 4.11. Three-Dimensional Structure Analysis

The 3TPA.1 (*G. Parasuis* 29755 strain) template in Protein Data Bank (PDB) was selected for *G. parasuis* HbpA protein epitope analysis. The results were generated using the PyMOL molecular visualization system.

### 4.12. Statistical Analysis

The experiments were repeated three times. All statistical data were analyzed by GraphPad Prism version 7.0 and expressed as mean ± SD. The differences among the truncations of the HbpA protein were analyzed using one-way ANOVA. Statistical changes were marked by **** *p* value < 0.0001.

## 5. Conclusions

In this study, rHbpA protein was successfully expressed, and three hybridoma cells (named 5D11, 2H81, and 4F2, respectively) against HbpA protein were produced and screened. Among the three hybridoma cells, 5D11 had the highest binding affinity with the HbpA protein and was chosen for further epitope mapping. The minimal epitope (aa325-PQYEFNLEKAKALLA-339, named EP-5D11) recognized by mAb 5D11 was defined as the highly conserved linear B-cell epitope. Therefore, mAb 5D11 and its defined linear epitope EP-5D11 could be used for applied research associated with *G. parasuis* diagnosis.

## Figures and Tables

**Table 1:** Primers used in this study.

Segment	Primers	Primer Sequences (5′→3′)	Positions (Amino Acids)
HbpA	HbpA-F	cagcaaatgggtcgcggatccGCACCGACAAATACATTGGTCA	1–510
	HbpA-R	ctcgagtgcggccgcaagcttTTAAGGCTTCAGACTTACGCCAT	
HbpA1	HbpA1-F	gccatggctgatatcggatccGCACCGACAAATACATTGGTCA	1–101
	HbpA1-R	ctcgagtgcggccgcaagcttGCGCTTGGCGTTGGAATGA	
HbpA2	HbpA2-F	gccatggctgatatcggatccGCCGATGATGTGGTGTTCTC	90–197
	HbpA2-R	ctcgagtgcggccgcaagcttGGTGGTCGGTCTGATAAGTTTGG	
HbpA3	HbpA3-F	gccatggctgatatcggatccCAACCGATTGGAACGGGG	181–286
	HbpA3-R	ctcgagtgcggccgcaagcttGTCGGCGAACTTTTACGTTGTTT	
HbpA4	HbpA4-F	gccatggctgatatcggatccGGGCTAAATACCACCAAACCTG	271–380
	HbpA4-R	ctcgagtgcggccgcaagcttGCACGCCAATTTTAGCCCAG	
HbpA5	HbpA5-F	gccatggctgatatcggatccATTCAAGCAGACTGGGCTAAAA	317–450
	HbpA5-R	ctcgagtgcggccgcaagcttGTTGTACCGCTGAGGCGAGTAG	
HbpA6	HbpA6-F	gccatggctgatatcggatccACCAATTACTCTCGCTGGACAG	431–510
	HbpA6-R	ctcgagtgcggccgcaagcttGAGGCTTCAGACTTACGCCATAA	
HbpA4-1	HbpA4-1-F	gccatggctgatatcggatccGGGCTAAATACCACCAAACCTG	271–318
	HbpA4-1-R	ctcgagtgcggccgcaagcttGTAATACTGCATCAGGGAACGGA	
HbpA4-2	HbpA4-2-F	gccatggctgatatcggatccGCAACCAATCCGTTCCCTG	309–348
	HbpA4-2-R	ctcgagtgcggccgcaagcttGTTCAAAACCGTTTGGATAGCC	
HbpA4-3	HbpA4-3-F	gccatggctgatatcggatccAAAGCATTATTGGCAGAAGCTG	335–380
	HbpA4-3-R	ctcgagtgcggccgcaagcttGCACGCCAATTTTAGCCCAG	
HbpA4-2-1	HbpA4-2-1-F	gccatggctgatatcggatccGCAACCAATCCGTTCCCTG	309–329
	HbpA4-2-1-R	ctcgagtgcggccgcaagcttGAAATTCATATTGTGGCAAATGC	
HbpA4-2-2	HbpA4-2-2-F	gccatggctgatatcggatccTATAACCCGCATTTGCCACA	320–339
	HbpA4-2-2-R	ctcgagtgcggccgcaagcttGTGCCAATAATGCTTTTGCTTTT	
HbpA4-2-3	HbpA4-2-3-F	gccatggctgatatcggatccAACTTGGAAAAAGCAAAAGCATT	330–348
	HbpA4-2-3-R	ctcgagtgcggccgcaagcttGTTCAAAACCGTTTGGATAGCC	
HbpA4-2-4	HbpA4-2-4-F	gccatggctgatatcggatccGCAACCAATCCGTTCCCTG	309–334
	HbpA4-2-4-R	ctcgagtgcggccgcaagcttGTGCTTTTTCCAAGTTAAATTCATAT	
HbpA4-2-5	HbpA4-2-5-F	gccatggctgatatcggatccTTGCCACAATATGAATTTAACTTGG	324–348
	HbpA4-2-5-R	ctcgagtgcggccgcaagcttGTTCAAAACCGTTTGGATAGCC	

Restriction endonuclease sites: BamHI and HindIII (underlined). Lowercase letters are homologous recombination fragments required for in-fusion cloning to the adjoining segments or vectors.

## Data Availability

The accession number of the *hbpA* gene of *G. parasuis* is CP001321.1. The PDB ID of the HbpA protein of *G. parasuis* is 3TPA.
